# Use of the World Health Organization’s Medical Eligibility Criteria for Contraceptive Use Guidance in sub-Saharan African Countries: A Cross-Sectional Study

**DOI:** 10.9745/GHSP-D-16-00216

**Published:** 2016-09-28

**Authors:** Melissa J Chen, Mary E Gaffield, James Kiarie

**Affiliations:** a University of California, Davis Department of Obstetrics and Gynecology, Sacramento, CA, USA; b World Health Organization, Department of Reproductive Health and Research, Geneva, Switzerland

## Abstract

Given recent updates to the postpartum contraception recommendations in the fifth edition of the *Medical Eligibility Criteria for Contraceptive Use* (MEC), published by the World Health Organization (WHO), the purpose of this qualitative study was to assess the extent to which national family planning policies in sub-Saharan African countries are in agreement with the WHO MEC, particularly with regard to postpartum contraceptive use. WHO headquarters sent questionnaires to country-level focal points to complete with their Ministry of Health counterparts. Between February and May 2016, 23 of 32 (72%) surveys were completed. All respondents reported that their countries had used the MEC document in the past, with most reporting that they had used the guidance as a reference (n = 20, 87%), for training purposes (n = 19, 83%), to change clinical practices (n = 17, 74%), and to develop national policies (n = 16, 70%). While many respondents (16, 70%) indicated their countries already include immediate postpartum intrauterine device insertion among breastfeeding women in their family planning policies, few reported currently allowing use of progestogen-only pills (n = 8, 35%) or implants (n = 8, 35%) during the immediate postpartum period (i.e., less than 48 hours after delivery) for breastfeeding women. A higher percentage of respondents indicated their countries allowed breastfeeding women the option of progestogen-only pills (n = 16, 70%) and implants (n = 13, 57%) between 48 hours and 6 weeks postpartum. Findings from this baseline assessment suggest that many countries may benefit from training and policy formulation support to adapt both new WHO MEC updates as well as existing recommendations from previous MEC revisions into national family planning guidelines.

## Abstract (precis)

The revised 2015 World Health Organization guidance expanded the recommended contraceptive options available to breastfeeding women during the early postpartum period to include progestogen-only pills and implants, but a substantial number of surveyed country representatives indicated that as yet their national policies did not allow such women to use these methods at that time. Countries may benefit from support to incorporate MEC guidance into national service delivery guidelines.

## INTRODUCTION

High unmet need for postpartum contraception exists globally despite renewed focus on postpartum family planning.^1^ Lactating women experience contraceptive effects while breastfeeding; however, this protection remains only when the woman is exclusively breastfeeding, amenorrheic, and within 6 months postpartum.^2^ Up to 62% of all women who gave birth in the last year have an unmet need for contraception—that is, they do not want to become pregnant within the next 2 years but are not using contraception—according to survey data from 57 countries.^1^ The highest rates are among women who live in West and Central Africa (75%) and East and Southern Africa (65%).^1^ A greater understanding of the barriers to postpartum contraception is needed to address these health care gaps.

The World Health Organization (WHO) published the first edition of the *Medical Eligibility Criteria for Contraceptive Use* (MEC) in 1996 to provide evidence-based guidance on which clients can safely use various contraceptive methods.^3^ WHO has updated this guidance periodically based on the latest scientific evidence and is currently in its fifth edition (published in 2015).^4^ The goal of the guidance is to improve the quality of family planning care and expand access to contraceptive use worldwide. In the guidance document, an eligibility category from 1 to 4 is assigned to a health condition or characteristic to indicate the degree of restriction for use of a particular method in the presence of that specific health condition or characteristic (Table). The target audience of the MEC includes policy makers and family planning program managers, and the intention is for the MEC document to serve as a reference for each individual country’s adaptation into national service delivery guidelines and policies.

The updated 2015 WHO Medical Eligibility Criteria expanded the contraceptive options for postpartum women, including breastfeeding mothers, to encompass POPs, implants, and the levonorgestrel-releasing IUD.

**TABLE:** World Health Organization *Medical Eligibility Criteria for Contraceptive Use* Categories for Contraceptive Eligibility

Category	Definition	Interpretation	
		With Good Resources for Clinical Judgment	With Limited Resources for Clinical Judgment
1	A condition for which there is no restriction for the use of the contraceptive method	Use method in any circumstances	Use the method
2	A condition where the advantages of using the method generally outweigh the theoretical or proven risks	Generally use the method	
3	A condition where the theoretical or proven risks usually outweigh the advantages of using the method	Use of method not usually recommended unless other more appropriate methods are not available or not acceptable	Do not use the method
4	A condition which represents an unacceptable health risk if the contraceptive method is used	Method not to be used	

In the most recent edition of the MEC, WHO convened meetings of a Guideline Development Group consisting of a wide range of stakeholders to review 14 topics. The updated recommendations have expanded the contraceptive options for postpartum women, including breastfeeding mothers, in an effort to increase access to suitable methods without compromising safety from adverse events or complications from contraceptive use.

In this article, we focus on the recent MEC changes related to contraceptive eligibility for postpartum women, which include^5^:

1. A change from WHO MEC category 3 (use of the method not usually recommended unless other more appropriate methods are not available or not acceptable) to category 2 (generally use the method) for use of progestogen-only pills (POPs) and implants among breastfeeding women who are less than 6 weeks postpartum
2. A change from category 3 to category 2 for use of the levonorgestrel-releasing intrauterine device (IUD) among breastfeeding women who are less than 48 hours postpartum
3. Additional risk stratification for venous thromboembolism for use of combined hormonal contraceptives (CHCs) among non-breastfeeding women based on time since delivery: those who are less than 21 days postpartum (category 4, method not to be used) and those who are 21 days postpartum or more (category 3)

In early 2016, we conducted a qualitative study to assess the extent to which national family planning policies in sub-Saharan Africa are in agreement with the 2015 WHO MEC. In this article, we focus particularly on the agreement between the national policies and the MEC regarding the use of various contraceptive methods during the postpartum period and focus specifically on African countries because of the particularly high unmet need among postpartum women identified in surveys.

## METHODS

Between February and May 2016, WHO headquarters sent baseline evaluation questionnaires (either in English or French, depending on the country) to 32 focal points in WHO country offices within sub-Saharan Africa (see supplementary material for English version of the questionnaire). The surveys asked about the countries’ family planning policies in terms of which contraceptive methods can be provided to women who are (1) postpartum and breastfeeding, (2) younger than 20 years old, (3) nulliparous, or (4) living with HIV infection and taking antiretroviral therapy. In addition, the questionnaire included items regarding emergency contraception policies and risky sexual behavior. The country-level focal points were asked to complete the survey in conjunction with their national Ministry of Health counterparts to increase accuracy of reporting.

We made 3 e-mail requests to complete the surveys over a period of 12 weeks. The WHO Ethics Review Committee considered this quality assessment study, with data reported in the aggregate and not by individual country, exempt from full review.

## RESULTS

Of the 32 country-level focal points, 23 completed the survey (72% response rate). Responding countries are listed in the Box. While most respondents (n = 19, 83%) reported that their countries have used the MEC document more than 2 times in the past, 4 (17%) respondents reported that their countries used the MEC document only 1 to 2 times. The government has the responsibility of distributing family planning guidance in most countries (n = 14, 61%); in other cases, WHO country offices or NGOs perform distribution. Respondents indicated that the MEC is primarily used in their respective countries as a reference (n = 20, 87%), for training purposes (n = 19, 83%), to change clinical practices (n = 17, 74%), and to develop national policies (n = 16, 70%).

BOX **List of Countries That Completed the Questionnaire in Alphabetical Order**

- Benin
- Burundi
- Chad
- Comoros
- Congo
- Côte d’Ivoire
- Democratic Republic of Congo
- Gabon
- Ghana
- Kenya
- Lesotho
- Liberia
- Madagascar
- Mali
- Mozambique
- Namibia
- Niger
- Nigeria
- Rwanda
- Senegal
- Tanzania
- Uganda
- Zimbabwe

With regard to the immediate postpartum period (i.e., less than 48 hours after delivery), 16 (70%) countries include IUD insertion as a contraceptive option. In contrast, few countries currently incorporate POPs (n = 8, 35%) or implants (n = 8, 35%) in their guidelines as methods eligible for breastfeeding women during the immediate postpartum period.

70% of respondents indicated their countries allowed POP use among breastfeeding women between 48 hours and 6 weeks postpartum and 57% allowed implant use.

Among breastfeeding women who are between 48 hours and 6 weeks postpartum, respondents indicated that POP and implant use are included in the national policies of 16 (70%) and 13 (57%) of their countries, respectively. Ten (44%) respondents indicated their countries also allow depot medroxyprogesterone (DMPA) and norethindrone enanthate (NET-EN) injections for breastfeeding women who are less than 6 weeks postpartum, which has a category 3 classification in the MEC.

Among breastfeeding women who are greater than 6 weeks postpartum, 6 (26%) countries allow CHC use. The MEC classifies breastfeeding women who are between 6 weeks and 6 months postpartum as category 3 for use of CHCs, whereas breastfeeding women who are 6 months or more postpartum are classified as category 2. All other methods (i.e., POPs, DMPA, NET-EN, implants, and the IUD) are included in national policies, as recommended in the MEC.

## DISCUSSION

This qualitative assessment reveals that there is a need to improve awareness in sub-Saharan African countries that POPs and implants can be used during the first 6 weeks postpartum to expand contraceptive options for breastfeeding women. Evidence suggests that early initiation of POPs or implants does not adversely affect breastfeeding performance, maternal health, or infant growth parameters; review of this evidence resulted in the change from category 3 to category 2 in the updated WHO MEC guidance.^4^

There is an opportunity to improve awareness in sub-Saharan African countries that POPs and implants can be used during the first 6 weeks postpartum to expand contraceptive options for breastfeeding women.

In addition, while many sub-Saharan African countries include immediate postpartum IUD insertion in national family planning guidelines, our findings indicate that this practice has not been universally adopted by all countries despite the introduction of this recommendation in the fourth (2009) edition of the WHO MEC.^6^ Although the priority for WHO and implementing partners is to support countries in adapting the *most recent* WHO MEC updates into national family planning guidelines, some countries may also benefit from technical review of current policies to incorporate existing recommendations from *previous* WHO MEC revisions.

Countries may benefit from technical review of their national family planning guidelines to incorporate both the most recent WHO MEC updates and previous MEC revisions.

Our findings also revealed that some country guidelines are more lenient than the WHO MEC recommendations for certain methods and conditions. For example, the WHO MEC recommendations remain unchanged for DMPA and NET-EN use among breastfeeding women who are less than 6 weeks postpartum (category 3) due to theoretical concern of neonatal exposure to steroid hormones. However, almost half of the survey respondents reported the inclusion of DMPA or NET-EN as a contraceptive option for this group of women in their national guidelines. It is possible that individual countries consider the benefits of contraceptive access outweigh the potential harm, thus leading to this difference in national policies from WHO guidance. Similarly, some countries reported allowing use of CHCs among breastfeeding women after 6 weeks postpartum, whereas the WHO MEC recommends allowing use of the method among this group of women after 6 months postpartum. These countries may consider that access to CHCs outweighs the potential risks of shortened breastfeeding duration due to decreased milk supply with estrogen exposure. Family planning guidelines in other countries, such as the United States^7^ and United Kingdom,^8^ are also more lenient on these issues, allowing for earlier use of DMPA and NET-EN injectables as well as CHCs among breastfeeding women. These differences reinforce WHO’s intention that the MEC guidance serve as a framework for each country to adapt in accordance with local needs, values, preferences, and resources.

More than 50 national programs across the world have adopted the WHO MEC guidance.^9^ In addition to updating and distributing the MEC recommendations, WHO and implementing partners can provide technical assistance to countries who request training in the MEC guidance. Findings from this study provide an initial assessment of areas for further education among country policy makers, program managers, and service providers. Many factors contribute to contraception initiation and continuation, especially in the postpartum setting, but having comprehensive, up-to-date, and evidence-based policies is the first step for countries to improve access to postpartum contraception and reduce unmet need for their citizens.

### Limitations

This study was based on a self-reported survey of national policies, and not an independent review of the policies, which may lead to reporting bias. In addition, the country-level focal points were instructed to complete the survey in conjunction with their Ministry of Health counterparts; however, some surveys were completed either by the Ministry of Health personnel or WHO country-level focal points and not by both parties together, potentially affecting the accuracy of the responses. Despite follow-up requests from WHO headquarters, 9 (28%) country-level focal points did not complete the survey. A non-response bias may be present if some countries did not respond to the survey because attention is being shifted away from family planning services. Additionally, the survey was available in French and English, and only one of two invited Lusophone (Portuguese-speaking) countries participated.

## CONCLUSION

This qualitative assessment revealed a need to support country policy makers on incorporating the updated WHO MEC recommendations into national guidelines, particularly with regard to expanding the contraceptive options available to breastfeeding women during the early postpartum period. Future assessments can explore reasons for the discrepancies between MEC guidelines and national family planning policies to guide efforts in training and policy formation.

## Supplementary Material

supplementary material

## Notes

**Peer Reviewed**
