# Supplementary material for: Use of the World Health Organization’s Medical Eligibility Criteria for Contraceptive Use Guidance in sub-Saharan African Countries: A Cross-Sectional Study
Source: Glob Health Sci Pract. 2016 Sep 28;4(3):506–10. doi: 10.9745/GHSP-D-16-00216 (PMC5042704; doi:10.9745/GHSP-D-16-00216)
Supplement: supplementary material [file GHSP-D-16-00216_index.html]

Supplement to Use of the World Health Organization’s Medical Eligibility Criteria for Contraceptive Use Guidance in sub-Saharan African Countries: A Cross-Sectional Study | Global Health: Science and Practice

## GHSP-D-16-00216 Supplementary Material

Chen et al. doi: 10.9745/GHSP-D-16-00216

- Supplementary Material - Chen et al. doi: 10.9745/GHSP-D-16-00216
