# Supplementary material for: Use of the World Health Organization’s Medical Eligibility Criteria for Contraceptive Use Guidance in sub-Saharan African Countries: A Cross-Sectional Study
Source: Glob Health Sci Pract. 2016 Sep 28;4(3):506–10. doi: 10.9745/GHSP-D-16-00216 (PMC5042704; doi:10.9745/GHSP-D-16-00216)
Supplement: supplementary material [file 16-00216-Chen-Supplementary-material.pdf]

General information

1. Country: ____

2. Email address: _____________________________ (for contact purposes, in case of questions)

3. What is your country's experience with the document, *Medical eligibility criteria for contraceptive use* (check all responses that apply)

□ No prior knowledge □ Have seen but not used
□ Have used 1-2 times □ Have used more than 2 times

(If the MEC has been used, please respond to questions 4 and 5, otherwise, skip to question 6.)

4. How has the MEC guideline been used (check all responses that apply)?

□ For reference □ To develop a specific strategy □ To develop national policy
□ To change practices □ For advocacy for family planning □ For training purposes
□ Other: ____

5. Is the MEC document distributed by the government in your country?

□ Yes □ No, if so by who? __________________________________

Information on national family planning/contraceptive policies

6.) According to national policies, which contraceptive method or methods can be provided to women who are **postpartum** and **breastfeeding** ? (check all boxes that apply.)

Timing	Combined Pills	Progestogen-only pills	DMPA or NET-EN injection	Implants	Condoms	Intrauterine Devices	None
Immediately after delivery of the placenta to < 48 hours							
48 hours to < 6 weeks postpartum							
6 weeks to < 6 months postpartum							

7.) According to national policies, which contraceptive method or methods can be routinely offered to young women (< 20 years) ? (check all boxes that apply.)

	Pills ‡	Injections	Implants	Condoms	Intrauterine Devices	None
Young women, < 20 years						
Married, young women (< 20 years)						

‡ Includes both combined (estrogen-progestogen) pills and progestogen-only pills

8.) According to national policies, which contraceptive method or methods can be provided to women with the following conditions/characteristics (check all boxes that apply):

	Combined Pills	Progestogen-only pills	DMPA or NET-EN injection	Implants	Condoms	Intrauterine Devices	None
Women living with HIV infection							
Women taking antiretroviral therapy (ART)							
Nulliparous women$^{\phi}$							

$^{\phi}$ Regardless of age and/or marital status

9.) According to national policies, individuals reporting risky sexual behaviour and requesting contraception are counselled to (check all responses that apply):

□ Use condoms □ Use condoms consistently and correctly
□ Use an effective method of contraception □ Use condoms or another preventive measure
□se condom and additional method of contraception
□ Follow other advice (name): ____

10.) According to national policies, which emergency contraception methods are offered (check all responses that apply)?

□ Emergency contraceptive pills (ECP)
→ If ECP is offered, which regimens are included in the national policy ?
___ combined oral contraceptive pills ___ levonorgestrel pills ___ ulipristal acetate pills

□ Emergency intra-uterine device (E-IUD)

□ Emergency contraception is not included in the national policy

<u>Thank you for participation ! Please feel free to provide other comments</u>

____
____
____
____
____
____
